# Febrile neutropenia in children and adolescents with acute myeloid leukemia during induction therapy

**DOI:** 10.3389/fped.2026.1883346

**Published:** 2026-07-22

**Authors:** Manel Rebah, Emna Azza, Emna Ben Jemia, Yosr Ben Abdennebi, Eya Ben Othmen, Roua Hsasna, Maroua Bahri, Lamia Aissaoui

**Affiliations:** 1 Clinical Hematology Department, Oncohematology UR14ES11, Aziza Othmana Hospital, Faculty of Medicine of Tunis, University of Tunis El Manar, Tunis, Tunisia; 2 Pulmonology Department, Charles Nicolle Hospital, Faculty of Medicine of Tunis, University of Tunis El Manar, Tunis, Tunisia

**Keywords:** acute myeloid leukemia, children, febrile neutropenia, infection, mortality

## Abstract

**Background:**

Febrile neutropenia (FN) is a serious and potentially life-threatening complication in children with acute myeloid leukemia (AML), particularly during induction chemotherapy. However, data from low- and middle-income countries remain limited.

**Methods:**

We conducted a retrospective study including children and adolescents with newly diagnosed AML who experienced at least one FN episode during induction therapy at Aziza Othmana Hospital, Tunis, Tunisia.

**Results:**

Among 63 patients with AML, 55 (87.3%) experienced 82 FN episodes. No patient received antibacterial or antifungal prophylaxis. Clinically documented infections (CDI) accounted for 53.6% of episodes, fever of unknown origin for 25.7%, and microbiologically documented infections (MDI) for 20.7%. The gastrointestinal tract was the most frequent site of infection (50%), followed by the respiratory tract (31.8%). Blood cultures yielded predominantly Gram-negative bacilli and Gram-positive cocci in similar proportions. Multidrug-resistant Gram-negative bacteria were identified in five episodes. Clinical outcomes were favorable in 77 episodes (94%), with a median time to defervescence of four days. Five patients died, resulting in a FN-related mortality rate of 9%. Intensive care unit (ICU) admission and presence of sepsis/septic shock were associated with mortality in univariate analysis.

**Conclusion:**

FN remains a major cause of morbidity and mortality during AML induction therapy. Early recognition, prompt antimicrobial treatment, and improved access to microbiological diagnostics may improve outcomes in resource-limited settings.

## Introduction

Febrile neutropenia (FN) is defined as fever accompanied by neutropenia, most commonly occurring in patients undergoing chemotherapy for cancer. It is a serious complication of acute myeloid leukemia (AML) treatment, occurring in more than 80% of patients during the first course of chemotherapy (1). This is primarily due to prolonged neutropenia induced by induction therapy (2). The mortality associated with this complication is approximately 9.3% (3). However, a remarkable decrease in mortality has recently been observed, attributed to the use of broad-spectrum antibiotics and advances in supportive care (4). A better understanding of the infectious causes of FN could help optimize the management of diagnostic and treatment delays, as prompt initiation of adequate antimicrobial therapy within one hour is recommended (5, 6). FN may be of unknown origin in a variable proportion of cases, but it can also be clinically or microbiologically documented. Therapeutic management relies on broad-spectrum antibiotic therapy, either prescribed empirically or guided by the clinical focus and/or microbiological documentation.

The profile of infections during FN remains poorly elucidated in pediatric populations, with conflicting findings across studies (7).

The aim of our study was to describe the clinical, biological, and evolutionary profile of FN episodes in children and adolescents newly diagnosed with AML during the induction phase of chemotherapy as well as to find risk factors associated with FN mortality.

## Patients and methods

We conducted a retrospective study that included children and adolescents treated for AML in Aziza Othmana Hospital (Tunisia) and diagnosed with FN during the induction phase according to the World Health Organization (WHO) classification and risk stratification based on cytogenetic and molecular abnormalities (8).

### Inclusion and exclusion criteria

All patients aged < 18 years with newly diagnosed AML who received induction chemotherapy between January 2016 and December 2025 and experienced at least one FN episode during the induction phase were included. Patients with secondary AML, relapsed AML, or those who did not complete induction therapy were excluded.

Patients were treated according to one of the following induction protocols: AIDA, AML02, 3 + 7.

- The **AIDA** protocol consisted of Idarubicin 12 mg/m²/day×4 days + ATRA 25 mg/m²/day administered in two divided doses until remission for acute promyelocytic leukemia (APL) (9).
- The AML02 protocol, which comprised Mitoxantrone 12 mg/m²/day×5 days in combination with Cytarabine 200 mg/m²/day×7 days, was the standard of treatment for non-APL (10).
- The **3** **+** **7** regimen was administered using either Idarubicin or Daunorubicin in cases where Mitoxantrone was unavailable. It consisted of 3 days of Idarubicine or Daunorubicine combined with 7 days of Cytarabine 200 mg/m²/day (11).

Demographic data, clinical characteristics, laboratory results, antibiotic treatments, severe complications, and mortality data were collected from the medical records of the patients.

**FN** was defined as a single oral temperature of ≥38.3 °C or a temperature of ≥38 °C sustained over 1 h in patients who had an absolute neutrophil count (ANC) of <500/mm^3^ or ANC between 500 and 1,000 expected to decrease below 500/mm^3^ within 48 h (5). A new episode was defined as a fever occurring after at least 72 h of afebrile status. One patient may have one or more episodes of FN.

**Clinically documented infection (CDI)** was defined by the presence of clinical symptoms or radiological evidence of infection without microbiological confirmation. **Microbiologically documented infection (MDI)** was defined by the presence of microbiological evidence.

**Enterocolitis:** Is defined by one clinical symptom or sign, and confirmatory imaging showing cecum wall thickness >3 mm on ultrasonography (12).
**Determination of gastrointestinal source of bacteremia:** The gastrointestinal tract was considered the source of bacteremia when a patient presented at least one of the following clinical findings within 48 h of a positive blood culture: abdominal pain, diarrhea (≥3 loose stools per day), nausea or vomiting, or abdominal tenderness on physical examination

Multidrug-resistant Gram-negative bacteria were defined as isolates showing non-susceptibility to at least one agent in three or more antimicrobial classes.

Coagulase-negative staphylococci were considered true pathogens only when isolated from at least two separate blood cultures in the presence of compatible clinical findings; otherwise, they were considered contaminants.

The normal laboratory range for C-reactive protein (CRP) was < 5 mg/L.

### Statistical analysis

Statistical analyses were performed using SPSS version 22.0. Continuous variables were expressed as medians with ranges. Categorical variables were expressed as frequencies and percentages. The association between each potential risk factor (pulmonary infection, intensive care unit admission, microbiological documentation, ANC <100/mm³, age ≥15 years, presence of sepsis/septic shock) and mortality was assessed using Fisher's exact test or chi-square test for categorical variables. Overall survival according to ICU admission or sepsis/septic shock was estimated using the Kaplan–Meier method, and curves were compared using the log-rank test. A *p*-value <0.05 was considered statistically significant.

## Results

Among 63 patients with AML, 55 (87.3%) were included with a median age at diagnosis of 9 years (range: 2–17), and a male to female ratio of 1.5. The characteristics of patients at diagnosis are shown in Table 1.

**Table 1:** Patient characteristics at diagnosis.

Category	Clinical feature	Number (*N* = 55)
Sex	Male	33
	Female	22
Age	2–14 years	39
	≥15 years	16
Risk group	Favorable risk	21
	Intermediate risk	14
	Unfavorable risk	14
	Unknown	6
Protocol treatment	AML02	26
	AIDA	12
	Induction 3 + 7	17

A total number of 82 FN episodes was recorded, with a median of one (range:1–3) episodes. Median ANC rate was 124/mm^3^ (range: 0 to1000). Median value of C-reactive protein (CRP) was 34 mg/L (3–423). CRP was above 90 in 14 episodes and was associated with microbiologically documented infection (MDI) FN (*p* < 0.001).

- FN with clinically documented infection CDI was the most frequent (53.6%) followed by fever of unknown origin (FUO)(25.7%) and MDI (20.7%) (Table 2).
- There were three cases in which more than two clinical foci were found. In one case, the foci were located in the digestive system and the otorhinolaryngological area. In the remaining two cases, the foci involved both pulmonary and mucosal sites. Organisms were isolated by blood cultures in 14 cases, from anal swab tests in two cases identifying oxyuriasis, and by *Aspergillus* antigen testing in one case. Among MDI, Gram-negative bacilli (GNB) and Gram-positive cocci (GPC) accounted for five isolates each, representing similar proportions of bacterial pathogens. The gastrointestinal tract was the source of bacteremia in 4 of 10 cases (40%). Five isolates fulfilled criteria for multidrug-resistant GNB. Fungi (4.8%) were also a prominent cause of infections with Trichosporon identified in three cases.

**Table 2:** Clinically or microbiologically documented infection.

Documented FN episodes	Number (%)
MDI: *n* = 17 (20.7%): Gram positive cocci: Coagulase-negative staphylococci (contaminants) Enterococcus faecium Enterococcus faecalis Gram negative bacilli: Enterobacter cloacae Serratia marcescens Acinetobacter baumannii Klebsiella pneumoniae Fungus: Trichosporon spp. Candida albicans Aspergillus Parasite: Enterobius vermicularis	3 (17.6%) 1 (5.9%) 1 (5.9%) 1 (5.9%) 1 (5.9%) 2 (11.7%) 1 (5.9%) 3 (17.6%) 1 (5.9%) 1 (5.9%) 2 (11.8%)
CDI: *n* = 44 (53.6%): Gastro-intestinal tract Cutaneomucous Respiratory tract Urinary Otorhinolaryngology	22 (50%) 9 (20.4%) 14 (31.8%) 1 (2.2%) 2 (4.4%)

FN, febrile neutropenia; MDI, microbiologically documented infection; CDI,clinically documented infection.

Among FN episodes, initial treatment involved antibiotic monotherapy in 45 cases, mainly with a *β*-lactam agent. Bitherapy or triple therapy was used in 30 episodes, combining a *β*-lactam with a glycopeptide and an aminoglycoside. Antifungal therapy was introduced in 7 cases following antibiotic treatment in previous episodes.

Directed antibiotic therapy based on antibiogram results was administered in 6 episodes. Antifungal therapy was administered in 48 FN episodes. Targeted antifungal treatment was prescribed in seven episodes with documented fungal infection, including invasive aspergillosis (*n* = 4) and fungemia (*n* = 3). In addition, empirical antifungal therapy, predominantly amphotericin B, was initiated in 41 episodes because of persistent fever despite broad-spectrum antibacterial treatment. Septic shock and sepsis occurred in 4 and 3 cases, respectively. Regarding ICU indications, four patients were transferred to the ICU.

The outcome was favorable in 77 episodes (94%) with a median time of defervescence of 4 days (range:1–22). Five patients died primarily due to FN (9%) (three because of respiratory distress due to sepsis and two because of septic shock) with a median time from diagnosis of 4 days (range: 1–29).

**Figure 1:**
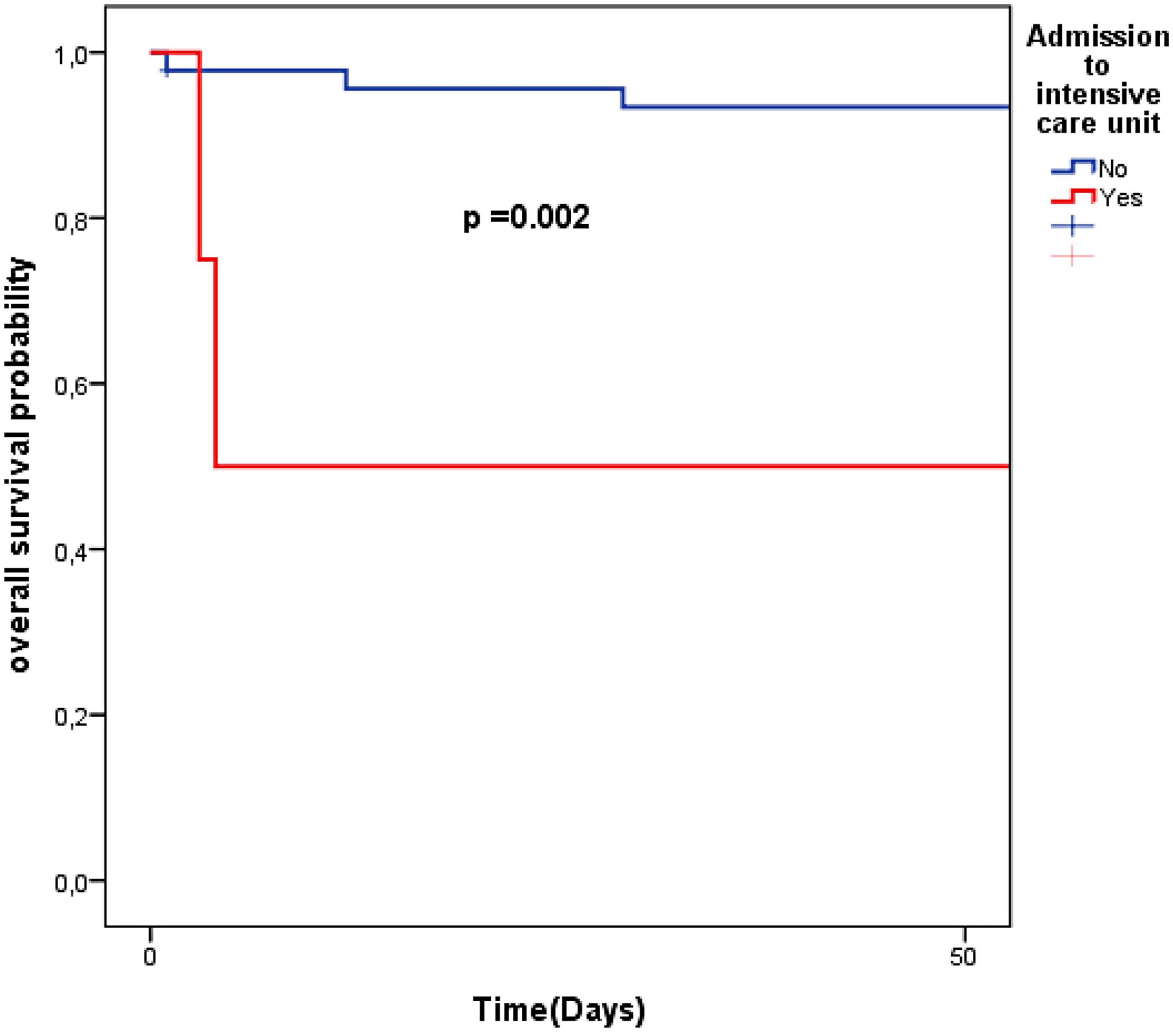
Overall survival according to transfer to intensive care unit.

**Figure 2:**
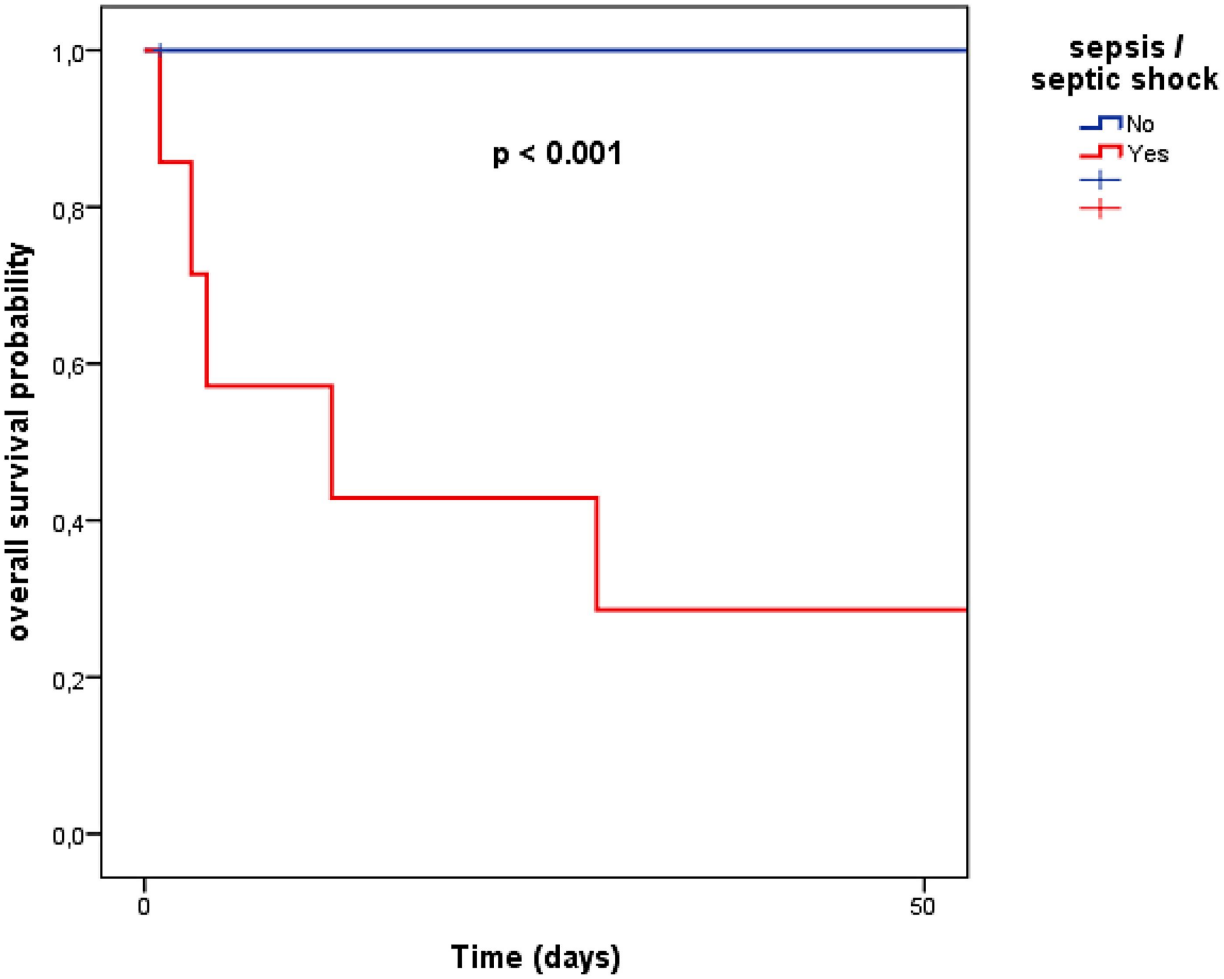
Overall survival according to the presence of sepsis/septic shock.

In univariate analysis, only admission to the ICU and presence of sepsis or septic shock were significantly associated with mortality (Figures 1, 2; Table 3).

**Table 3:** Risk factors for febrile neutropenia mortality.

Factor	Percentage of mortality related to FN	*P*
Age: ≥15 years vs. <15 years	8.3% vs. 9.3%	1
APL vs. Non APL	9.1% vs. 9.1%	1
PNC: <100/mm3 vs. ≥100/mm3	5.3% vs. 10%	1
High risk vs. low or intermediate risk	7.1% vs. 9.8%	1
Pneumonia vs. without pneumonia	50% vs. 11.1%	0.092
MDI vs. non MDI	0% vs. 11.1%	1
Admission to ICU vs. non admission	50% vs. 4.4%	0.029
Septic shock/sepsis versus without	71.4% vs. 0%	*P* < 0.001

FN, febrile neutropenia; APL, acute promyelocytic leukemia; PNC, polynuclear neutrophil count; MDI, microbiologically documented infection.

## Discussion

This study aimed to characterize the clinical and microbiological landscape of FN episodes in a cohort of pediatric patients with AML during the induction phase.

Our results provide a view of the infectious complications that occur during this critical period, revealing that CDI was the most frequent, representing 53.6% of FN episodes. The most frequent site was gastrointestinal (GI) tract, with approximately 22 cases (50%), followed by pulmonary (27.5%) and mucocutaneous sites (20.5%). This was confirmed in various international studies. According to Parikh et al. (2018), during the induction phase of AML, the GI tract was the most commonly identified source, accounting for 45.6% of documented infection sites. Similarly, Lakshmaiah et al. (2015) reported that in high-risk patients with hematological malignancies, the GI tract was the predominant focus, implicated in 46.6% of cases where a clinical site of infection could be identified (13, 14).

Radiologically documented enterocolitis was observed in 8 cases. This could be explained by the frequency of diarrhea, which may have multiple causes during induction, as well as by the sterilization of enteral feeding and gastrointestinal decontamination practices varying between countries. Our results align with those of a prospective study by Castagnola et al. (2010), who reported that in pediatric AML patients, the GI tract was the source of bacteremia in over 40% of FN episodes (15).

Blood cultures yielded predominantly GNB and GPC in similar proportions. This equal distribution contrasts with the predominance of GPC reported in Western countries, where central line-associated bloodstream infections are more common. The lack of accessibility to central venous catheters in our setting likely reduces the incidence of these device-related infections, thereby revealing a more balanced microbiological profile.

The rate of MDI in our study (20.7%) is notably lower than other studies as for the landmark multi-institutional AML-BFM 93 trial by Lehrnbecher et al., which documented a rate of 32.1% across 855 infectious episodes in 304 pediatric AML patients (16). The low proportion of MDI in our series (19.5%) could be explained by the inadequacy of the infectious disease investigation and the difficulty in accessing several microbiological tests in our country, and particularly in our hospital: a low number of sputum cultures performed, occasional shortages of sputum collection containers, and a lack of respiratory multiplex PCR, among others.

Routine colonization screening was not performed; however, colonization was documented in two patients, both with extended-spectrum beta-lactamase producing *E. coli*. Some studies have shown that Colonization with multidrug resistant organisms determines the clinical course of patients with AML undergoing intensive induction chemotherapy. It also can be associated with longer durations of hospitalization and a higher 30-day mortality rate (17). A landmark study by Sung et al. (2007), which analyzed data from over 400 children with AML treated on a multi-institutional North American trial, found that while Gram positive organisms were responsible for the majority of bloodstream infections (55%), Gram-negative organisms accounted for a substantial 38% of cases. Importantly, they also reported that GNB infections were associated with a significantly higher risk of infection-related mortality (18).

Concerning fungal infections, according to Cannas et al., Nunez et al., and Lappalainen et al., the prevalence of fungal infections was approximately 9%, 7%, and 2.3%, respectively (19, 20). In our study, we have identified fungemia for trichosporon in three cases, candida albicans in one case and aspergillus in one case (6%).

According to infectious diseases society of America guidelines (IDSA), prophylactic measures are indicated for neutropenic patients. Prophylaxis with fluoroquinolones is recommended only in cases of neutropenia associated with a high risk of infection. The study reports that, during chemotherapy courses with prophylaxis combining teicoplanin and ciprofloxacin, the incidence of FN was 43% (21). Antifungal prophylaxis against Candida and Aspergillus is also recommended for patients undergoing intensive chemotherapy for AML, in whom the risk of invasive fungal infections is substantial (21). During the study period, prophylactic antimicrobial agents were not routinely administered in our institution because of local practice patterns, concerns regarding antimicrobial resistance, and limited access to certain drugs. These factors may have contributed to the high incidence of FN observed in our cohort.

We found that there is an association between a CRP above 90 and the documentation of FN episodes. This was confirmed by a study by El-Maghraby et al. (2007) that evaluated the diagnostic value of CRP in 85 FN episodes occurring in 76 children with hematologic malignancies which find that high CRP help diagnose infectious episodes (22).

The first-line antibiotic therapy in our department was a monotherapy targeting GNB, particularly covering Pseudomonas Aeruginosa, in accordance with international guidelines (5). This was also consistent with The International Pediatric Fever and Neutropenia Guideline who recommends initial empiric monotherapy with an antipseudomonal beta-lactam (23).

Nevertheless, it must also be tailored to the specific context of each department and the patient's clinical data (24). The administration of Aminoglycosides and glycopeptides was not standardized in our department. Nevertheless, the role of Aminoglycosides is clearly defined in U.S. and European guidelines (5). According to the IDSA, isolated, unexplained and persistent fever in a stable patient is not an indication for escalating treatment. However, empirical antifungal therapy is indicated in high-risk patients if no source of the fever has been identified after four to seven days of broad-spectrum antibiotic therapy. In our study, Amphotericin B was administered empirically 72 h after the start of initial antibiotic therapy in cases of isolated persistent fever.

The difficulty in accessing intensive care represents a major problem for hematology in Tunisia. The timing of the transfer also plays an important role. Delays in care are implicated in the progression to death. Mortality related to FN was observed in 5 cases (9%).

ICU admission and the presence of sepsis or septic shock were significantly associated with mortality in our cohort. However, this finding should be interpreted with caution, as only four patients required ICU transfer. ICU admission likely reflects greater clinical severity rather than serving as an independent predictor of death. Furthermore, potential delays in ICU transfer may have contributed to this association. Although mortality was numerically higher among patients with pneumonia, this association did not reach statistical significance. According to Azerefegne et al, several independent predictive factors significantly influence 30-day mortality in patients with FN secondary to hematologic disorders. The study identifies that the presence of profound neutropenia (ANC < 100/mm^3^) and shock at the time of presentation are strongly associated with increased mortality (25). Another study from Turkey analyzed 49 pediatric AML patients and their survival outcomes and found that patients with intermediate or high risk had shorter overall survival than low-risk patients (26).

While many of our individual findings, such as GI tract predominance and the association between ICU admission and sepsis/septic shock and mortality, have been reported previously, our study provides several unique contributions. First, to our knowledge, this is the largest description of FN during AML induction therapy in a North African pediatric population. Our cohort reflects a different reality: no routine prophylaxis, limited access to central lines, and restricted diagnostic resources. Second, the equal distribution of Gram-positive and Gram-negative pathogens observed in our study contrasts with the Gram-positive predominance reported in Western series, and this difference is directly attributable to the low use of central venous catheters in our setting, a finding that has practical implications for empiric antibiotic selection in similar resource-limited environments. Third, our low rate of MDI (20.7%) quantifies the diagnostic gap that exists when advanced microbiological tests are unavailable, serving as a benchmark for quality improvement efforts in low- and middle-income countries. Finally, the high incidence of FN (87.3%) in the absence of prophylaxis provides real-world data that can inform decisions about implementing prophylactic strategies in settings where these are not yet routine. Thus, while our study confirms many known epidemiological patterns, it uniquely demonstrates how these patterns manifest and how clinical management must adapt when resources are constrained.

## Conclusion

This study provides one of the few descriptions of FN during AML induction therapy in a North African pediatric population. Unlike several Western series reporting a predominance of Gram-positive infections, we observed a similar distribution of Gram-positive and Gram-negative pathogens together with a predominance of GI infectious foci. The study also highlights the challenges encountered in resource-limited settings, including restricted access to microbiological investigations, limited screening for multidrug-resistant organisms, and the absence of routine antimicrobial prophylaxis.

## Data Availability

The raw data supporting the conclusions of this article will be made available by the authors, without undue reservation.
